# Kutane Muzinose der Kindheit

**DOI:** 10.1007/s00105-020-04743-8

**Published:** 2020-12-22

**Authors:** Lisa Eholzer, Ilske Oschlies, Mark Berneburg, Sigrid Karrer

**Affiliations:** 1 Klinik und Poliklinik für Dermatologie, Universitätsklinikum Regensburg, Franz-Josef-Strauß-Allee 11, 93042 Regensburg, Deutschland; 2 Institut für Pathologie, Sektion Hämatopathologie und Lymphknotenregister, Universitätsklinikum Schleswig-Holstein, Kiel, Deutschland

**Keywords:** Kinder, Seltene Erkrankungen, Kutane Papeln, Plaques, Histologie, Children, Rare diseases, Cutaneous papules, Plaques, Histology

## Abstract

Die kutane Muzinose der Kindheit ist eine sehr seltene Hauterkrankung mit nur wenigen beschriebenen Fällen in der Literatur. Wir berichten über einen 11-jährigen Jungen mit seit 9 Monaten bestehenden, asymptomatischen, hautfarbenen Papeln und Plaques am rechten Arm. Histologisch zeigten sich dermal und tief dermal zwischen den Kollagenbändern ausgeprägte Muzinablagerungen und Fibroblastenproliferationen. Da es sich bei der kutanen Muzinose der Kindheit um eine benigne Erkrankung mit guter Prognose handelt, ist eine Therapie nicht notwendig.

## Abstract (en)

Cutaneous mucinosis of infancy is a rare skin disease with just a few reported cases in the literature. We report the case of an 11-year-old boy with asymptomatic, skin-coloured papules and plaques on his right arm that had appeared 9 months prior to presentation. Histology showed a dermal and deep dermal interstitial mucin deposition and fibroblast proliferation. However, because cutaneous mucinosis of infancy is a benign disease with a good prognosis, therapy is not mandatory.

## Anamnese

Ein 11-jähriger Junge stellte sich in unserer ambulanten Sprechstunde vor. Er berichtete, dass es seit etwa 9 Monaten sukzessive zur Entstehung von nunmehr 6 symptomlosen Knötchen am rechten Arm gekommen sei. Es seien keine Vorerkrankungen bekannt. Medikamente werden nicht eingenommen. Die Familienanamnese sei hinsichtlich eines Lupus erythematodes bei der Großmutter des Jungen positiv.

## Befund

**Figure 1:**
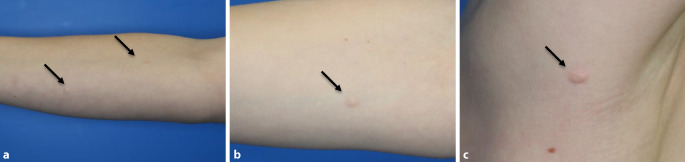

Am rechten Arm sowie axillär rechts zeigten sich insgesamt 6 derbe, exophytische, hautfarbene, maximal 0,8 cm durchmessende Papeln und Plaques, vereinzelt mit Teleangiektasien (Abb. 1a–c). Das restliche Integument erschien unauffällig.

## Diagnose

**Figure 2:**
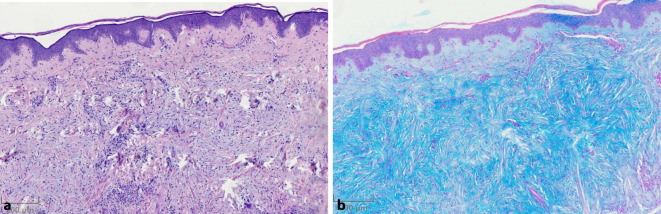

In einer Biopsie aus einer Papel vom rechten Oberarm zeigte sich histologisch eine minimale reaktive Akanthose der Epidermis. Dermal und tief dermal fand sich zwischen den Kollagenbändern eine deutliche Muzinose, die auch in der Alcianblau-Färbung sehr gut zur Darstellung kam (Abb. 2a, b). Die Muzinose war in allen Abschnitten diffuser Natur, zusätzlich diskrete periadnexielle lymphoplasmazelluläre Infiltrate, zwischen den Kollagenfasern Fibroblastenproliferationen. Atypische oder pleomorphe Zellen waren nicht nachweisbar. Nach dermato- und paidopathologischem Konsil sowie unter Berücksichtigung der klinischen Präsentation wurde die Diagnose einer kutanen Muzinose der Kindheit gestellt.

## Therapie und Verlauf

Aufgrund der Symptomlosigkeit und Harmlosigkeit der Hautveränderungen und da bei dem Patienten kein Therapiewunsch bestand, wurde auf eine Behandlung verzichtet.

## Diskussion

Die kutane Muzinose der Kindheit ist eine sehr seltene Erkrankung, die mit asymptomatischen lokalisierten Papeln und Plaques einhergeht. Sie wurde 1980 erstmals von Lum beschrieben, der bei einem 16 Monate alten Kleinkind einzigartige klinische und histologische Veränderungen feststellte, die nicht in die Gruppe der bekannten Muzinosen einzuordnen waren [3]. Seither wurden nur sehr wenige Fälle in der Literatur beschrieben. Die kutane Muzinose der Kindheit ist neben der diskret papulösen Muzinose, der akral persistierenden papulösen Muzinose, der selbstheilenden juvenilen kutanen Muzinose und der nodulären Muzinose eine von insgesamt 5 Subtypen der lokalisierten papulösen Muzinosen. Diese stellt wiederum eine der 3 Unterformen der idiopathischen primären kutanen Muzinose dar: generalisierte papulöse und sklerodermiforme Läsionen, lokalisierte papulöse Läsionen und Mischform [1]. Generell sind von den kutanen Muzinosen v. a. Erwachsene und nur selten Kinder betroffen. Die Tab. 1 gibt daher eine Übersicht über die primär kutanen Muzinosen, die im Kindesalter auftreten können.

**Table 1:** 

Muzinose	Klinik	Histologie	Therapie	Prognose	Systembeteiligung
**I. Degenerativ/entzündlich**					
*A. Dermal/subkutan*					
Kutane Muzinose der Kindheit	Hautfarbene/gelbliche/rötliche Papeln und Plaques an Stamm und Extremitäten, bei Geburt vorhanden oder Beginn in der Kindheit	Dermale interstitielle Muzinablagerungen, geringe Fibroblastenproliferation	Nicht notwendig	Gut, spontane Rückbildung möglich	Keine
Selbstheilende juvenile kutane Muzinose	Akute Eruption von hautfarbenen Papeln an Händen, Kopf, Stamm und Extremitäten und subkutane Knoten am Kopf und periartikulär, manchmal nach Infektion des oberen Respirationstraktes, Beginn im Alter von 1 bis 15 Jahren	Dermale Muzinablagerungen mit geringer Fibroblastenproliferation und perivaskuläre lymphozytäre Infiltrate, in den subkutanen Knoten zudem Infiltrate aus plumpen Spindelzellen und epitheloide Riesenzellen in myxoidem Stroma	Symptomatisch mit Glukokortikoiden	Gut, spontane Rückbildung innerhalb von Wochen bis Monaten	Meist Fieber, Arthralgien, Schwäche, Muskelschmerzen, Blutbildveränderungen
Sklerödem (postinfektiöser Typ)	Indurierte Ödeme im Nacken und am oberen Rücken, Auftreten nach fieberhaftem Infekt, meist vor dem 20. Lebensjahr	Stark verbreiterte Dermis mit verdickten, teils fensterartig auseinandergedrückten Kollagenbündeln, dazwischen Muzinablagerungen	Infektsanierung	Spontane Rückbildung innerhalb von einigen Monaten bis 2 Jahren	Möglich
Atypische papulöse Muzinose der Kindheit	Einzelne oder konfluierende Papeln, bei Geburt vorhanden oder Beginn bis zum 14. Lebensjahr	Dermale Muzinablagerungen mit variabler Fibroblastenproliferation	Symptomatisch	Nicht vorhersagbar	Keine oder variable Begleitsymptome
*B. Follikulär*					
Follikuläre Muzinose Pinkus (Alopecia mucinosa)	Einzelne bis wenige Alopezieherde, follikulär gebundene Papeln und Knoten, follikuläre Keratosen, akneiforme Läsionen am Kopf/Nacken, Auftreten bei Kindern und jungen Erwachsenen	Muzinablagerungen innerhalb des Follikelepithels und der Talgdrüsen, kein Epidermotropismus atypischer Lymphozyten	Keine	Spontane Rückbildung innerhalb von 2 Monaten bis 2 Jahren	Keine (bei älteren Patienten Assoziation mit T‑Zell-Lymphomen!)
**II. Hamartomatös/neoplastisch**					
*A. Dermal*					
Muzinöser Nävus	Hautfarbene bis gelblich-bräunliche Papeln oder Plaques, in lineärer oder Blaschko-lineärer oder gruppierter Anordnung am Rücken. Bei Geburt vorhanden oder Beginn im Jugendalter	3 Typen: Bindegewebstyp: Muzin in der papillären Dermis mit Verlust von Kollagen- und elastischen Fasern Kombinierter Typ: epidermaler Nävus mit Muzinablagerungen in der papillären Dermis Perifollikulärer Typ: Muzin in der papillären Dermis und um dilatierte Follikel	Exzision, Laserabtragung nur beim kombinierten Typ	Gutartig	Keine
Muzinöser ekkriner Nävus	Einzelner erythematöser oder bräunlicher Knoten oder Plaque an den Beinen oder multiple Läsionen in lineärer Anordnung oder entlang der Blaschko-Linien oder bilateral. Lokalisierte Hyperhidrose. Beginn meist vor der Pubertät, selten bei Geburt oder im Erwachsenenalter	Lobulär angeordnete ekkrine Schweißdrüsen und Gänge in der retikulären Dermis, die von reichlich Muzin umgeben sind, milde Fibroplasie	Exzision, intraläsionale Steroide, Behandlung der lokalisierten Hyperhidrose	Gutartig, keine Regressionstendenz	Keine
Superfizielles Angiomyxom (kutanes Myxom)	Einzelner, 1–5 cm großer Knoten am Stamm, Kopf, Nacken oder Bein. Multiple Läsionen im Gehörgang oder periorbital möglicherweise Hinweis auf Carney-Syndrom. Jeweils Beginn in der Kindheit möglich	Lobuläre Läsion mit myxoider Matrix in der Dermis und Subkutis mit prominenter Vaskularisation, plump spindeligen und sternförmigen Fibroblasten, Mastzellen und wenigen Kollagenfasern	Komplette Exzision	Einzelne Läsionen gutartig, bei Carney-Syndrom Herzbeteiligung mit embolischen Komplikationen	Bei Carney-Syndrom zusätzlich Lentigines, blaue Nävi, kardiale Myxome
*B. Follikulär*					
Nävoide follikuläre Muzinose	Multiple lineäre fleischfarbene, konfluierende Papeln entlang der Blaschko-Linien im Gesicht, Stamm, Armen. Bei Geburt vorhanden. Nur 1 Fall in der Literatur beschrieben!	Muzinablagerungen innerhalb des Follikelepithels und der Talgdrüsen	Keine	Gutartig	Keine

Klinisch manifestiert sich die kutane Muzinose der Kindheit mit asymptomatischen, meist 2–5 mm und maximal 20 cm durchmessenden, hautfarbenen bis weißlichen oder leicht erythematösen bis bräunlichen, manchmal gruppierten oder linear angeordneten Papeln und Plaques, die bevorzugt am Stamm, an den Extremitäten und seltener auch im Gesicht, am Kopf und am Hals auftreten [1, 10]. Während unser Patient die Entstehung der Läsionen erst ab dem 11. Lebensjahr bemerkt hat, treten die papulösen Läsionen laut Literatur meist in der frühen Kindheit bis spätestens zum 2. Lebensjahr auf, und häufig bestehen diese bereits seit Geburt [2, 5, 6, 8]. Verläufe mit an Anzahl und Größe progredienten Hautläsionen sind beschrieben, allerdings gibt es auch Fälle mit spontaner Rückbildung im Laufe der Pubertät [1, 6, 7]. Bei einer Langzeitbeobachtung einer Patientin mit bereits seit Geburt bestehender kutaner Muzinose der Kindheit am Oberschenkel kam es bis zum Alter von 2 Jahren zum Auftreten multipler weiterer größenprogredienter Plaques am Stamm und an den Extremitäten. Danach bildete sich zunächst die kongenitale Plaque am Oberschenkel spontan zurück, und bis zum Alter von 7 Jahren hatten sich alle Läsionen unter Hinterlassung lediglich einiger atropher Papeln am Stamm zurückgebildet [6]. Insgesamt ist daher von einer guten Prognose der Erkrankung mit der Möglichkeit einer spontanen Rückbildung auszugehen.

Differenzialdiagnostisch ist bei Papeln und Plaques im Kindesalter an Neurofibrome, Fibrome, Dermatofibrome, juvenile Xanthogranulome, Bindegewebsnävi, dermale Nävi, Mastozytome oder Leiomyome zu denken. Auch der Lupus erythematodes tumidus kann im Kindesalter auftreten und zu erythematösen Plaques ohne epidermale Beteiligung führen, die histologisch ausgeprägte interstitielle Muzinablagerungen aufweisen, aber auch perivaskuläre und periadnexielle lymphozytäre Infiltrate [11].

Die Histologie bestätigt in Zusammenschau mit der Klinik die Diagnose einer kutanen Muzinose der Kindheit und zeigt ausgeprägte dermale Muzinablagerungen zwischen den Kollagenfasern mit meist nur geringer Proliferation von Fibroblasten und ohne wesentliche entzündliche Infiltrate. Assoziierte Komorbiditäten wie eine monoklonale Gammopathie beim Skleromyxödem oder Schilddrüsenerkrankungen beim prätibialen Myxödem sind bei den betroffenen jungen Patienten in der Literatur nicht beschrieben. Wie auch bei unserem Patienten ist ein Fallbericht publiziert, bei dem die Großmutter des Patienten an einem Lupus erythematodes litt [4]. Bei einem anderen 5‑jährigen, ansonsten gesunden Patienten mit kutaner Muzinose war in der Familienanamnese ein Morbus Basedow bekannt, bei dem es zu Ablagerungen von Mukopolysacchariden an verschiedenen Organen kommt [1].

Die Pathogenese der kutanen Muzinose der Kindheit ist weiterhin unklar. Normalerweise werden Proteoglykane (Muzin) nur in geringen Mengen von dermalen Fibroblasten produziert. Es wird angenommen, dass die abnorme Ablagerung von Muzin durch eine Aktivierung von Fibroblasten durch Autoantikörper, monoklonale oder polyklonale Immunglobuline, proinflammatorische Zytokine, virale Infektionen oder auch durch einen gestörten Muzinabbau zustande kommt [9]. Bei den idiopathischen Muzinosen der Kindheit ohne Systembeteiligung wäre eine Induktion der Muzinproduktion durch Infekte oder sonstige, bislang unbekannte Triggerfaktoren denkbar. Da auch Fälle mit familiärer Häufung beschrieben wurden, wird zudem ein genetischer Hintergrund diskutiert.

Aufgrund der guten Prognose ist keine Therapie notwendig. Es wurden jedoch Therapieversuche mit topischen Glukokortikoiden sowie Calcineurininhibitoren mit unterschiedlichem Erfolg beschrieben [9].

## Fazit für die Praxis

- Die kutane Muzinose der Kindheit ist eine sehr selten beschriebene Hauterkrankung, bei der es zum Auftreten von symptomlosen Papeln und Plaques kommt.
- Die Diagnose wird in Korrelation mit der Klinik histologisch bestätigt.
- Eine Therapie ist aufgrund der Harmlosigkeit der Hautveränderungen nicht notwendig.
